# Synergistic Approaches to Foodborne Pathogen Control: A Narrative Review of Essential Oils and Bacteriophages

**DOI:** 10.3390/foods14091508

**Published:** 2025-04-25

**Authors:** Rafail Fokas, Nikolaos Giormezis, Apostolos Vantarakis

**Affiliations:** 1 Department of Public Health, Medical School, University of Patras, 26504 Patras, Greece; 2 Department of Microbiology, Medical School, University of Patras, 26504 Patras, Greece; giormenik@upatras.gr

**Keywords:** essential oils, bacteriophages, antimicrobial activity, foodborne pathogens, synergy

## Abstract

The emergence of antimicrobial resistance among foodborne pathogens has intensified the search for alternative biocontrol strategies. Among these, essential oils (EOs) and bacteriophages have gained increasing attention, due to their natural origin and antimicrobial potential. This narrative review investigates their individual and combined use as innovative tools for improving food safety. We discuss the mechanisms of action, current food applications, and regulatory or technical limitations associated with both EOs and phages. Particular emphasis is placed on their complementary characteristics, which may enhance efficacy when used together. An in-depth analysis of five key studies investigating synergistic EO–phage combinations against *Staphylococcus aureus*, *Escherichia coli*, and *Salmonella Typhimurium* is presented. These studies, conducted in both in vitro and food-based systems, reveal that antimicrobial synergy is often dose- and temperature-dependent. Optimized combinations lead to enhanced bacterial reduction and reduced resistance development. However, several challenges remain, including sensory alterations in food products, phage inactivation by EO compounds, and host cell destruction at high EO doses. The review concludes that while EOs and phages face limitations when applied independently, their strategic combination shows substantial promise. Future research should focus on formulation development, delivery systems, and regulatory alignment to unlock their full synergistic potential.

## 1. Introduction

Antimicrobial resistance (AMR) has emerged as a global crisis with profound implications for public health, food safety, and economic stability [1]. The increasing failure of antibiotics to effectively treat bacterial infections has resulted in prolonged illness, higher mortality rates, and escalating healthcare costs. In 2019, AMR was directly responsible for approximately 1.27 million deaths worldwide and contributed to an estimated 4.95 million deaths, making it one of the most urgent threats to modern medicine [2]. The crisis is largely attributed to the misuse and overuse of antimicrobials across multiple sectors, including human medicine, livestock farming, aquaculture, and crop production. This widespread reliance on antibiotics has accelerated the development of resistant bacterial strains, creating a pressing need for innovative solutions to mitigate this impact.

One of the most concerning aspects of AMR is its direct association with foodborne pathogens. The food industry plays a pivotal role in the emergence and dissemination of resistant bacteria, due to the extensive use of antibiotics in food production [3]. Antimicrobials are routinely administered in livestock and aquaculture, not only to treat infections, but also to promote growth and prevent disease outbreaks. This practice, while economically beneficial, has led to the emergence of multidrug-resistant (MDR) pathogens that can be transmitted to humans through the consumption of contaminated meat, dairy, and seafood. Foodborne bacteria such as *Salmonella* spp., *E. coli*, *Campylobacter* spp., and *Listeria monocytogenes* have shown increasing resistance to critical antibiotics [4], limiting treatment options and exacerbating the risk of foodborne illnesses. The economic and public health consequences of AMR are profound. Healthcare systems worldwide face mounting costs due to longer hospital stays and the necessity for more expensive and complex treatments. The World Bank estimates that AMR could result in global GDP losses of up to $3.4 trillion annually by 2030 [5], further underscoring the urgency of addressing this issue. In the food industry, AMR threatens production efficiency, increases the cost of regulatory compliance, and reduces consumer confidence in food safety. Contaminated food products not only endanger human health, but also contribute to trade restrictions and economic losses, particularly in regions heavily reliant on food exports. The interconnected nature of AMR means that addressing resistance in food systems is as crucial as controlling it in clinical settings. Despite global efforts to regulate antibiotic use and improve food safety practices, significant challenges remain in controlling AMR. The overuse of antibiotics in agriculture, particularly in regions with weak regulatory frameworks, continues to drive resistance [6]. The persistence of resistant bacteria in food processing environments and their transmission through global trade complicates containment efforts. Furthermore, the limited development of new antibiotics has created a critical gap in the fight against resistant infections [7]. Given these challenges, there is a pressing need to explore alternative and complementary antimicrobial strategies that can reduce reliance on antibiotics while effectively controlling foodborne pathogens.

Among the most promising non-antibiotic agents for use against resistant bacteria are EOs [8] and bacteriophages [9], which have demonstrated strong antimicrobial properties against a wide range of foodborne pathogens. EOs, derived from plants, contain bioactive compounds that disrupt bacterial membranes, inhibit quorum sensing, and reduce biofilm formation [10]. Their broad-spectrum antimicrobial activity makes them viable candidates for food preservation and infection control [11]. However, variations in their chemical composition, along with potential sensory modifications in food products and existing regulatory hurdles, pose challenges to their widespread adoption [12].

Bacteriophages, viruses that specifically infect and lyse bacteria, offer a highly targeted approach to pathogen control without affecting beneficial microbiota. Unlike antibiotics, phages evolve alongside bacteria, making it more difficult for pathogens to develop resistance [13]. Their use in food safety applications, including meat decontamination [14], dairy processing [15], and fresh produce preservation [16], has shown promising results in reducing bacterial contamination. However, concerns regarding phage stability in food matrices and regulatory approval must be addressed before they are integrated into commercial food production.

Essential oils can weaken bacterial defenses, increasing the susceptibility of pathogens to phage infection. In parallel, bacteriophages contribute precise targeting and lytic action against antibiotic-resistant bacteria. This dual approach not only enhances food safety, but also aligns with One Health principles, which emphasize the interconnectedness of human, animal, and environmental health. By leveraging the complementary mechanisms of these two antimicrobial agents, it may be possible to develop sustainable and effective alternatives to conventional antibiotics for use in food production.

The objective of this review is to explore the synergistic potential of essential oils and bacteriophages as alternative antimicrobial strategies to ensure food safety. Specifically, this review will examine the role of AMR in foodborne pathogens, the limitations of conventional antibiotics, and the effectiveness of essential oils and bacteriophages in controlling resistant bacteria. Furthermore, it will analyze the potential for synergy between these two approaches, and discuss the challenges associated with their practical implementation in food systems. By synthesizing current research and evaluating innovative interventions, this review aims to contribute to the ongoing efforts to combat AMR and enhance the safety and sustainability of global food systems.

## 2. Materials and Methods

A structured literature search was conducted across the PubMed, Scopus, and Google Scholar databases, aiming to identify scientific publications related to the antimicrobial application of essential oils and bacteriophages in food systems. A structured literature search was conducted using keyword combinations related to essential oils (e.g., “essential oils”, “plant-based antimicrobials”, “antimicrobial activity”), bacteriophages (e.g., “bacteriophages”, “phage therapy”, “food safety”), and their combined antimicrobial application (e.g., “essential oils AND bacteriophages”, “synergy”, “combined antimicrobial effect”).

The logical operators “AND” and “OR” were applied to refine the search results. The primary objective of the literature review was to collect data on the separate application of essential oils and bacteriophages as natural antimicrobials in food preservation, and, more importantly, to identify existing studies evaluating the synergistic potential of these two agents against foodborne pathogens. Articles that explored the agents’ mechanisms of action, practical applications in food matrices, and effectiveness against antibiotic-resistant bacteria were prioritized. This thematic categorization was adopted not only for clarity and organizational flow, but also because it helped to identify an important imbalance in the literature. While there is a significant body of work on the individual use of essential oils and bacteriophages, studies focusing on their synergistic application—particularly in real food matrices—remain scarce. This observation further highlights the need for an integrated analysis, as provided in this review.

Three researchers (R.F., N.G., and A.V.) independently screened the titles and abstracts of the retrieved publications. Subsequently, the full texts of eligible articles were evaluated to determine their relevance to the review objectives. Only articles published in peer-reviewed journals, written in English, and presenting original experimental data or structured reviews were included. Studies focusing exclusively on either essential oils or bacteriophages were categorized accordingly, while a separate category was created for studies investigating their combined use.

## 3. Essential Oils

### 3.1. Chemical Composition and Bioactive Compounds

EOs are complex mixtures of volatile and aromatic compounds, acting as plant-derived secondary metabolites with well-documented antimicrobial and antioxidant properties. These chemical substances are utilized by the plants themselves for various purposes, including defense against pathogens, attraction of pollinators, and interspecies communication [17]. Given their volatile characteristics, they are classified into many primary categories according to their chemical structure. These include phenolic chemicals, terpenes and terpenoids, and aldehydes and ketones, as well as alcohols and esters. The content of EOs varies based on the plant species, ambient circumstances, plant portion, and extraction process [18]. Among the most numerous and medically significant components are phenolic compounds, such as thymol, carvacrol, and eugenol, which are commonly found in oils like thyme, oregano, and clove [19]. Carvacrol and thymol are phenolic compounds with well-documented antimicrobial properties, and have been extensively investigated for their efficacy against a broad spectrum of microorganisms. These compounds have demonstrated significant inhibitory effects on clinically and food-relevant pathogens, including *E. coli*, *L. monocytogenes*, *S. enterica* subsp. [20], and *S. aureus* [21] organisms, associated with serious health risks and high prevalence in foodborne outbreaks. Another prominent group is monoterpenes and terpenoids, which include molecules such as limonene, linalool, and pinene [22]. These compounds are commonly present in citrus oils [23], lavender [24], and pine-derived oils, and are responsible not only for the characteristic aromas of these plants, but also for a range of functional properties. Aldehydes, such as cinnamaldehyde (the major component of cinnamon oil) [25] and citral (found in lemongrass) [26], are also frequently detected in EOs, and contribute significantly to their functional profile. Ιn addition, aliphatic and aromatic alcohols, such as geraniol (found in rose and palmarosa oil) [27], menthol (present in peppermint oil) [28], and borneol, are frequently reported as significant constituents of various essential oils. While EOs typically contain two to three major dominant constituents in high concentrations, they may also include dozens of minor components that can influence their overall properties through synergistic or modulatory effects. This chemical complexity is intricately connected to a range of biological processes, with key constituents often involved in mechanisms that underpin the antimicrobial function of essential oils.

### 3.2. Antimicrobial Mechanisms of Action of Essential Oils

**Figure 1:**
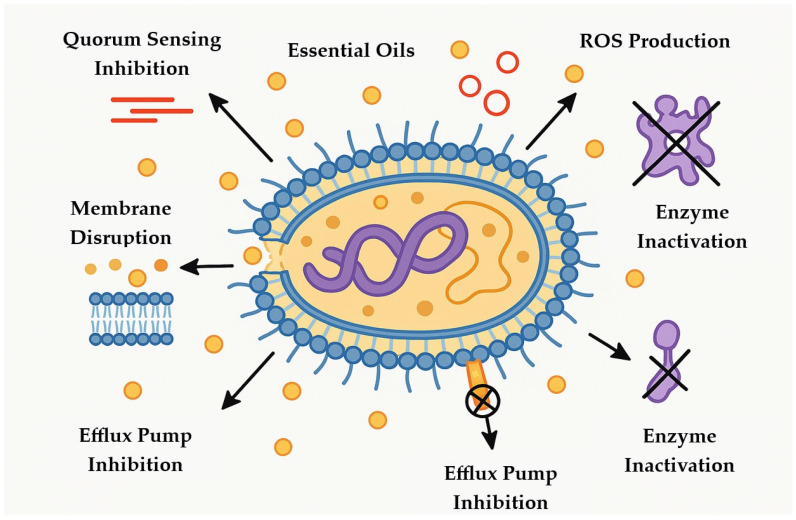
Antimicrobial mechanisms of essential oils against bacterial cells.

The antimicrobial activity of EOs results from the combined structural and functional damage they induce in microbial cells, mediated by various bioactive components [29]. Their intrinsic lipophilicity allows for integration into and rupture of bacterial cell membranes, resulting in enhanced permeability, leaking of intracellular contents, and the collapse of critical electrochemical gradients [30]. This membrane disruption not only compromises the structural integrity of bacterial cells, but also affects membrane-bound proteins that are essential for cellular function [10]. EOs also impair bacterial quorum sensing, a regulatory process necessary for biofilm formation and virulence factor expression, limiting bacteria’s capacity to build protective biofilms [31,32]. Furthermore, specific components have been shown to block bacterial efflux pumps, resulting in increased intracellular retention of antimicrobial drugs [33]. Finally, several EO ingredients generate reactive oxygen species (ROS), which cause oxidative stress and damage to proteins, lipids, and nucleic acids, eventually leading to bacterial cell death [34]. Collectively, these synergistic pathways (Figure 1) demonstrate EOs’ potential as efficient antibacterial agents against a wide range of foodborne pathogens. However, their efficacy can vary depending on food matrix composition, processing conditions, and environmental factors. Most mechanistic insights have been confirmed primarily in vitro, highlighting the need for further validation in complex food systems.

Thymol and carvacrol have hydroxyl groups that interact with bacterial membranes, causing membrane destabilization, disruption to proton gradients, and cytoplasmic leakage, ultimately leading to cell death [10]. Terpenes and terpenoids, such as limonene, linalool, and α-pinene, can modify membrane fluidity, increase permeability, interfere with ion transport channels, and inhibit enzymes involved in microbial metabolism and replication [35]. Cinnamaldehyde [36] and citral [37] have high antibacterial efficacy by attaching to microbial protein targets, inactivating enzymes and affecting cellular respiration. Cinnamaldehyde, a key component of cinnamon oil, effectively inhibits foodborne pathogens, including *L. monocytogenes* and *E. coli*, in food systems [38]. Alcohols and esters, such as geraniol [39], can impair membrane integrity and interfere with intracellular homeostasis, leading to antibacterial action. The overall performance of EOs is frequently attributed to synergistic or additive interactions between their major and minor constituents. The combination of thymol and carvacrol in oregano oil has been demonstrated to boost antibacterial activity as the two components potentiate each other’s actions on the bacterial membrane, causing more harm than when given singly [40]. The antimicrobial properties of EOs establish a strong foundation for their use in food preservation.

### 3.3. Applications of Essential Oils in Food Systems

EOs have gained popularity in the food business due to their robust antibacterial capabilities, which provide natural alternatives to synthetic preservatives. Their uses in food systems include a variety of approaches, each with unique benefits and limitations. Direct integration of EOs into food products can effectively suppress microbiological growth, extending their shelf life [41]. However, EOs’ strong smells and fragrances may affect the sensory properties of the food, and therefore, careful concentration optimization is needed to balance antibacterial efficacy and consumer acceptance [42]. To address this, EOs have been included into edible coatings and films used on food surfaces [43], forming barriers against microbial contamination while reducing sensory impact. This method has shown promise for preserving perishable products such as fruits, vegetables, and cheeses. Another novel application is active packaging solutions, which embed EOs in packaging materials, allowing for the controlled release of antimicrobial chemicals during storage and distribution [44]. This constant emission contributes to the long-term stability of food quality and safety.

### 3.4. Challenges and Limitations of EOs

Despite their potential antibacterial and antioxidant characteristics, the practical use of EOs in food preservation presents a number of obstacles and constraints. One major concern is their strong aroma and flavor, which can dramatically alter the sensory profile of food products, restricting consumer acceptability [45]. Furthermore, EOs are highly volatile and sensitive to degradation in the presence of light, heat, and oxygen, thereby reducing their efficacy during processing and storage [46]. Their interactions with food matrix components such as lipids, proteins, and carbohydrates can have a significant impact on their stability and antibacterial efficacy [47]. Regulatory systems introduce an additional layer of complexity, as permitted amounts and safety regulations differ between nations, necessitating strict compliance. Furthermore, establishing good antibacterial activity often requires relatively high concentrations of EOs, which may increase production costs, while also posing risks of toxicity or sensory rejection. Technically, homogeneous dispersion and controlled release of EOs in food systems remains a significant challenge. Given these limits, continuing research into encapsulation technologies and synergistic pairings with other preservation strategies intends to improve EO performance and viability in food applications. Although EOs have been extensively studied for their antimicrobial activity—both in food systems and via standardized protocols, such as those defined by EUCAST [48]—given the extensive existing literature and the well-documented nature of such studies, a detailed summary lies beyond the specific scope and focus of this review. Instead, innovation is likely to emerge through novel combinations of EOs with other antimicrobial agents, such as endolysins [49], bacteriocins [50], or bacteriophages [51]. These synergistic applications hold greater potential for overcoming current limitations, and may redefine the role of EOs in food preservation strategies. Among these strategies, the combination of EOs with bacteriophages stands out as particularly novel and underexplored. This emerging research direction appears to hold the greatest promise for innovation, offering an exciting area of development where the intersection of natural plant-based compounds and targeted viral therapies may redefine food safety interventions.

## 4. Bacteriophages

### 4.1. Biological Characteristics and Classification

Bacteriophages, often known as phages, are viruses that infect and replicate exclusively within bacterial cells. They are among the most abundant biological entities on the planet, helping to regulate bacterial populations in a variety of habitats [52]. Their distinct properties and many classifications have sparked substantial interest in microbiological study. Bacteriophage morphology is highly diverse, a feature that plays a critical role in both their classification and infection mechanisms. The most common form has a two-part structure: an icosahedral head (capsid) that contains the genetic material, and a tail that facilitates attachment to the bacterial host. This dual structure characterizes the order *Caudovirales*, which includes families such as *Myoviridae* (phages with contractile tails) [53]. *Siphoviridae* are phages with long, non-contractile tails [54], while *Podoviridae* have short, non-contractile tails [55]. Other morphological types include filamentous phages, such as those from the *Inoviridae* family, which have elongated, rod-like geometries [56]. Similarly, phages exhibit extensive genetic diversity. Their genomes can be made of DNA or RNA, single- or double-stranded, and range in size from a few to over a hundred kilobases. This genetic variation affects their replication tactics and interactions with host bacteria. Bacteriophage life cycles are divided into two categories: lytic and lysogenic [52]. During the lytic cycle, phages infect the host bacteria, hijack their cellular machinery to make more phage particles, and eventually cause the host cell to lyse in order to release progeny phages. During the lysogenic cycle, phage DNA integrates into the host’s genome, forming a prophage that replicates passively alongside the host cell’s DNA, without inflicting immediate harm [57]. Under specific circumstances, a prophage can reactivate and enter the lytic cycle. Understanding the biological properties and classification of bacteriophages is critical for realizing their potential in a variety of applications, including their use as natural antibacterial agents in food preservation. Their host specificity and capacity to control bacterial populations make them intriguing agents for treating bacterial infections.

### 4.2. Mechanisms of Antibacterial Action

**Figure 2:**
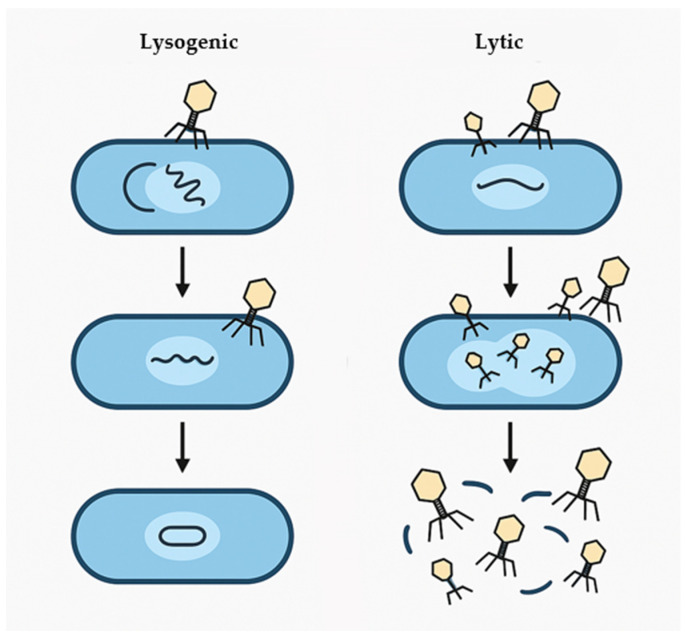
Lysogenic and lytic cycles of bacteriophages.

Bacteriophages exert their antibacterial effects via extremely precise biological mechanisms (Figure 2), which are predominantly triggered by the lytic infection cycle. This process starts with the phage adhering to the bacterial cell surface by very specialized interactions with receptors like lipopolysaccharides, teichoic acids, or membrane proteins [58]. These interactions determine the phage’s host range and play a critical role in its tailored antibacterial activity. Upon attachment, the phage injects its genetic material into the host cytoplasm, frequently using enzymatic processes to break the bacterial cell wall and allow DNA entrance [59]. Once it has entered the cell, the phage genome controls the bacterial metabolic apparatus, replicating its nucleic acids and synthesizing structural components. These components self-assemble into mature phage particles inside the host cell. The final step in the lytic cycle is the generation of lytic enzymes, predominantly endolysins, which break down the bacterial peptidoglycan layer, resulting in osmotic imbalance and cell lysis [60]. This causes the release of offspring phages, which can infect nearby bacterial cells, resulting in exponential proliferation at the site of infection. Unlike broad-spectrum antimicrobials, phages have a very specific mode of action, reducing collateral damage to beneficial bacteria and maintaining microbial equilibrium in complex contexts like food chains [13]. Furthermore, because they replicate themselves, they can remain active as long as vulnerable bacterial hosts are present. Beyond the lytic pathway, some phages can facilitate horizontal gene transfer by transduction, a process in which bacterial DNA is accidentally packed and transported across cells [61]. While this method promotes genetic variety, it may also introduce hazards, such as the spread of virulence or antibiotic resistance genes, if not well handled. Overall, bacteriophages’ precision, amplification capability, and flexibility make them intriguing agents for targeted bacterial control, particularly in applications requiring specificity and minimal disturbance to the microbial environment.

### 4.3. Applications of Bacteriophages in Food Systems

Bacteriophages are increasingly being used as natural biocontrol agents in food systems, due to their targeted specificity and ability to replicate themselves. Their ability to selectively lyse foodborne bacteria without disturbing the local microbiota or changing sensory properties has made them promising alternatives to traditional chemical preservatives. Bacteriophages can be used at many phases of the food supply chain, such as post-harvest treatment [62], surface processing [63], packaging, and direct application to food [64]. Several commercial phage-based products (Table 1) have been developed and licensed, most notably in the United States, where the Food and Drug Administration (FDA) has granted GRAS (Generally Recognized as Safe) designation to a variety of phage preparations targeting important foodborne pathogens [63]. Listex™ P100, a listeriophage preparation, targets *L. monocytogenes* in ready-to-eat (RTE) foods such meats, smoked salmon, and soft cheeses [65]. ListShield™, a GRAS-approved product, targets *L. monocytogenes* in processed meat and poultry products [66]. EcoShield™, a phage cocktail that targets *Escherichia coli O157:H7*, is used to treat red meat and beef carcasses [67]. SalmoFresh™, a preparation active against various *Salmonella* serotypes, is licensed for use in raw and cooked poultry, seafood, and produce [68]. The strategies for using bacteriophage products in food systems differ based on the food matrix and processing environment. These include surface spraying, immersion, incorporation into edible coatings, and use in antimicrobial packaging solutions. Phages have been demonstrated in studies to effectively lower pathogen burdens on animal-derived food products [69], but also on fruits and vegetables [70], especially when used under optimal conditions (e.g., proper time, pH, and temperature). Importantly, phages remain active on food surfaces and can reproduce in the presence of vulnerable bacterial hosts, providing a dynamic antimicrobial defense.

In the European Union, the regulatory landscape for phage-based food applications is less developed than in the United States (US) [71]. While no phage products have yet received full European Food Safety Authority (EFSA) approval for direct food use, several are being evaluated, and interest in phage biocontrol is growing among European Union (EU)-funded research projects. Bacteriophages are regulated under the EFSA Novel Food Regulation (EU 2015/2283), which requires the submission of extensive safety and efficacy data on a case-by-case basis [72]. Moreover, the absence of harmonized, phage-specific guidelines within the EU contributes to regulatory uncertainty and delays in commercial approval. In contrast, in the US, phage-based preparations such as Listex™ and Salmonelex™ have been granted GRAS status by the FDA, facilitating their use in food products. Despite regulatory disparities, the global spread of phage-based food technology is supported by an expanding body of peer-reviewed scientific evidence demonstrating their safety, specificity, and potential usefulness in reducing antibiotic-resistant bacteria in food supply chains. Overall, the incorporation of bacteriophages in food systems represents a practical, natural strategy for reducing contamination by pathogenic bacteria, with significant implications for food safety, shelf life extension, and public health.

**Table 1:** Summary of well-established approved bacteriophages for use in food products.

Product	Manufacturer	Target Pathogen(s)	Application Matrix	Regulatory
ListShield™ [73]	Intralytix Ltd. (Columbia, SC, USA)	*L. monocytogenes*	Ready-to-eat foods, non-food contact equipment, surfaces, etc., in food processing plants and other food establishments	FDA, 21 Code of Federal Regulations (CFR) 172.785; FDA, Generally Recognized as Safe Notice (GRN) 528; United States Environmental Protection Agency (EPA) Reg. No. 74234-1; Israel Ministry of Health; Health Canada
EcoShield™ [74]		*E. coli O157:H7*	Red meat surfaces	FDA, Food Contact Notification (FCN) 1018; Israel Ministry of Health; Health Canada
SalmoFresh™ [75]		*Salmonella* spp.	Poultry, fish and shellfish, and fresh and processed fruits and vegetables	FDA, GRN 435; United States Department of Agriculture (USDA), Food Safety and Inspection Service (FSIS) Directive 7120.1; Israel Ministry of Health; Health Canada
ShigaShield [76]		*Shigella* spp.	Food	FDA, GRN 672
PhageGuard Listex™ P100 [77]	Micreos Food Safety (Wageningen, The Netherlands)	*L. monocytogenes*	Cheese, fish, meat, ready-to-eat products	FDA, GRAS Notice (GRN) 198/218; Food Standards Australia New Zealand (FSANZ); EFSA; Swiss BAG; Israel Ministry of Health; Health Canada
PhageGuard S [78]		*Salmonella* spp.	Poultry, meat, cheese	FDA, GRN 468; FSANZ; Swiss BAG; Israel Ministry of Health; Health Canada
PhageGuard E [79]		*E. coli O157:H7*	Beef, vegetables	FDA, GRN 757
Salmonelex™ [80]		*Salmonella* spp.	Various foods	FDA and USDA, GRAS
Bafasal^®^ [81]	Proteon Pharmaceuticals (Mumbai, India)	*Salmonella* spp.	Animal feed, poultry farming	Approved in the EU

### 4.4. Challenges and Limitations of Bacteriophages

Despite their intriguing promise as natural antimicrobial agents in food systems, bacteriophages are faced with a number of problems and constraints that must be solved before they can be widely adopted in the industry. One of the most noticeable difficulties is their limited host range, which, while useful for targeting specific diseases without disrupting the native microbiota, limits their efficacy in complex microbial ecosystems [82]. Phages are very specialized, often infecting only one bacterial species or strain. As a result, phage preparations must be carefully chosen or synthesized as phage cocktails to widen their scope of action, increasing production complexity and cost. Another key restriction is the stability and durability of phages in food processing and storage environments. Τemperature, pH, moisture content, and the presence of inhibitory compounds in food matrices can reduce phage survival and infectivity [83]. For example, phages may become inactive in acidic environments or lose activity when exposed to thermal treatments [84], limiting their practical use in some food products unless protective formulations (e.g., encapsulation) are used. Furthermore, there is still a risk of bacterial resistance to phages. Although phages grow with their bacterial hosts and can eventually overcome resistance mechanisms, the emergence of phage-resistant variants can reduce efficacy, especially in long-term applications [85]. To address this, rotating phage use, phage adaptability techniques, and combination approaches with other antimicrobials are being studied. Regulatory hurdles also impede commercial implementation. Additionally, it is widely recognized that certain bacteriophages, particularly temperate phages, may promote horizontal gene transfer, including the mobilization of antibiotic resistance genes [86,87]. When phages are utilized inadvertently or without previous genomic screening, this type of broad or specialized transduction raises biosafety concerns. However, in food safety applications, such phages are rigorously avoided. Only virulent (lytic) phages are chosen for commercial or experimental applications [88], because they do not integrate into the bacterial genome, and so do not enable gene transfer. This intentional selection considerably minimizes the danger of unintended genetic spread and promotes the safe use of phage-based biocontrol in food systems. In spite of worries about the horizontal transmission of antibiotic resistance genes by temperate phages, there is also the possibility of bacterial resistance to phages themselves [85], especially if they are used repeatedly. This phenomenon, which has its roots in the ancient co-evolution of bacteria and phages, is mediated by mechanisms such as CRISPR-Cas systems, surface receptor changes, and restriction–modification processes [89]. Although resistance may develop, it can be reduced by using phage mixtures, rotation techniques, and combination therapies that maintain potency over time.

While the FDA has granted GRAS clearance to various phage-based medicines in the United States, regulatory frameworks in other nations, particularly of the European Union, are fragmented and underdeveloped. With a lack of standardized norms, manufacturers are faced with ambiguity regarding approval procedures, labeling regulations, and safety assessments. Finally, consumer perception and public approval can have an impact on the market adoption of phage technologies. Despite their natural origin and documented safety, the idea of applying viruses to food may generate concerns among customers who are unfamiliar with the scientific basis of phage therapy. Transparent communication, education, and regulatory approval are thus essential for establishing confidence [88].

## 5. Synergistic Applications of Essential Oils and Bacteriophages

### 5.1. Overview of Experimental Studies

The reason for combining EOs and bacteriophages stems from their complimentary antibacterial activities. EOs, particularly those containing phenolic components such as thymol, carvacrol, or eugenol, affect the integrity of bacterial membranes, increasing permeability and changing membrane potential [10]. This disruption not only reduces bacterial viability, but it may also enhance the entry or activity of bacteriophages or phage-derived lytic enzymes. In Gram-negative bacteria, the outer membrane acts as a physical barrier that limits endolysin penetration to the peptidoglycan layer, thereby reducing their lytic efficacy, unless aided by permeabilizing agents or membrane-disrupting compounds such as EOs [90]. Bacteriophages, on the other hand, replicate within vulnerable bacterial cells, providing great specificity and self-amplifying antimicrobial activity. However, their efficiency may be hampered by barriers to phage adsorption, modifications to bacterial surface receptors, or the presence of resistant or physiologically stressed subpopulations [85]. EOs may weaken or destabilize these populations, making them more vulnerable to phage attack. Furthermore, phages and essential oils may target various cellular processes or structures, enhancing the possibility of additive or synergistic bactericidal effects while decreasing the risk of resistance formation.

In recent years, a modest but growing body of study has investigated the combined use of EOs and bacteriophages (or phage-derived enzymes) as antimicrobial agents in food systems. Although the number of studies is currently limited, they provide useful insights into the possible synergistic or additive interactions between the two, especially in battling multidrug-resistant foodborne bacteria. Table 2 provides a comparative overview of studies, summarizing the pathogens, antimicrobial agents, food matrices, methods, and key findings associated with each.

Ghosh [91] explored the combined use of EO compounds and phage K against *S. aureus* in vitro and on raw chicken pieces. While in vitro assays showed promising inhibition, results on chicken meat revealed limited synergy, especially at refrigeration temperatures. The study emphasized the importance of optimizing delivery strategies to ensure consistent contact and effectiveness in real food matrices. In a follow-up paper, Ghosh et al. [92] examined the combination of pine-derived EO compounds (alpha-pinene, 3-carene, limonene) and phage K against *S. aureus* in vitro. While both antimicrobials exhibited individual inhibitory effects, combined treatments showed only partial enhancement, with strain-dependent variability. The study suggests additive effects, but emphasizes the need for further optimization to achieve consistent synergy.

Abdallah et al. [51] demonstrated a more robust synergistic effect between thyme EO (1%) and a lytic phage (vB_SauM_CP9) in reducing *S. aureus* counts on artificially inoculated chicken fillets. The combination achieved an 87.2% reduction in bacterial load after 120 min, significantly outperforming each agent alone.

Kim et al. [49] evaluated the synergy between selected EOs (carvacrol, eugenol, thymol, AITC) and a phage-derived endolysin (LysPB32) against *S. typhimurium* in vitro and in cooked ground beef. Carvacrol and eugenol combined with LysPB32 led to >2-log reductions in bacterial load in food matrices, and complete inhibition in vitro. The study confirms that essential oils enhance membrane permeability, restoring endolysin activity, and thus represent a promising approach for natural food preservation.

Finally, Elafify et al. [93] tested multiple EOs (cinnamon, thymol, clove) alongside a phage cocktail (MS2 + T7) against *E. coli ATCC 15597*. Their findings showed strong synergistic reductions of over 5-log CFU/mL and decreased resistance emergence, particularly in combinations involving cinnamon and thymol, indicating that the EO–phage pairing can enhance antimicrobial effectiveness while suppressing resistant subpopulations.

While these studies provide valuable insights into the synergistic action of EOs and bacteriophages, their effectiveness appears to vary significantly depending on the food matrix. In surface-exposed products like meats and fresh produce, their combined use has demonstrated efficient pathogen reduction due to accessible interaction points. Conversely, in more complex matrices such as dairy or acidic liquids, challenges like phage instability and EO interference with food components may diminish efficacy. These outcomes emphasize the need to tailor application strategies to specific food types to fully harness the benefits of this dual antimicrobial approach.

### 5.2. Limitations, Considerations, and Practical Implications

Despite the great potential of combining EOs and bacteriophages as antimicrobial agents in food systems, a number of crucial criteria must be carefully evaluated when assessing their practical relevance. While several studies have found synergistic or additive effects in the laboratory, these results are not consistently reproducible, and appear to be highly dependent on a variety of environmental and biological variables.

One significant drawback concerns the physicochemical interactions between EOs and bacteriophages. Lipophilic EO chemicals can reduce phage viability by altering viral capsid architecture or interfering with adsorption to host cells [94]. Furthermore, the intense bactericidal effect of some EOs, particularly at higher doses, may destroy susceptible bacterial hosts before phage replication can occur, negating the self-amplifying benefit of phage therapy. On the other hand, the synergistic studies mentioned above typically used EOs at low concentrations—often half or even one-quarter of their minimum inhibitory levels—in order to weaken the bacteria’s membrane integrity and physiological resilience, rather than kill them outright. This sublethal exposure increases bacterial sensitivity, while retaining structural characteristics necessary for phage attachment and DNA injection. In such cases, EOs act as adjuvants, weakening bacterial defenses, interrupting biofilm formation, or suppressing efflux processes, allowing phages to function more effectively. Finally, the timing, dose, and order of delivery are crucial factors in determining whether the interaction between phages and EOs will be synergistic, neutral, or antagonistic.

Additional problems include the impact of food matrix composition, pH, water activity, and antibiotic administration methods. Encapsulation techniques, sequential application, and microenvironment control may be required to ensure that both agents remain active and reach their destination. Regulatory and commercial constraints also exist, particularly in the EU, where phage-based products [95] are still not officially approved for widespread food usage, and EOs are limited by flavor, volatility, and maximum residue levels. Nonetheless, when correctly designed, the combination of EOs and bacteriophages presents a promising natural strategy for combating foodborne pathogens, including multidrug-resistant strains, without the need of synthetic preservatives or broad-spectrum antibiotics. This dual method can increase microbiological safety, extend shelf life, and meet customer demand for clean-label food goods. Moving forward, its success will be dependent on the development of tailored delivery methods, in vivo validations, and increased regulatory harmonization.

## 6. Research Gaps and Future Perspectives

Despite encouraging preliminary results, the application of EOs and bacteriophages in food systems is still underexplored, with considerable knowledge gaps and technological barriers. First and foremost, the existing body of literature is fragmented and lacks established approaches. Most studies differ greatly in terms of bacterial strains, phage types, EO components, concentrations, matrices, and environmental conditions, including pH and temperature. To enable meaningful cross-comparisons and meta-analyses, uniform experimental frameworks, including consistent use of synergy measurement methods (e.g., checkerboard assays, Fractional Inhibitory Concentration [FIC] indices), are clearly required [96]. Furthermore, whereas some in vitro studies indicate synergistic potential, few studies have tested this strategy in complex food matrices under realistic conditions [97]. The transfer from laboratory assays to in vivo or food system studies is critical for verifying efficacy and understanding the role of matrix interactions, microbial ecology, and sensory factors. Without such translational studies, the practical utility of EO–phage combinations remains uncertain. Another important area for future research is the development of better delivery systems. Co-formulating phages and EOs in encapsulation matrices, nanoemulsions, or active packaging could allow for controlled release, protection of sensitive components, and improved sequential action. Technologies such as multilayer edible coatings, temperature-responsive capsules, and lipid-based vesicles have the ability to distribute these compounds in a coordinated, food-safe manner [98,99]. Furthermore, mechanistic studies integrating omics technologies—such as transcriptomics, proteomics, and metagenomics—are urgently required to understand how combination treatments alter microbial physiology and population dynamics [100,101]. Such information could enable predictive modeling of phage–EO interactions and resistance development, eventually directing the rational design of synergistic therapies. Finally, regulatory and consumer acceptance constraints must be overcome. Bacterial phages have yet to acquire widespread regulatory approval for food applications in the EU [102], and EOs remain limited in flavor and residue. Public impressions of viruses and pungent natural substances in food may complicate marketing. Moving forward, interdisciplinary collaboration between microbiologists, food technologists, regulatory authorities, and behavioral scientists will be required to turn this novel antibacterial notion into a feasible, industry-ready solution.

## 7. Conclusions

The growing number of MDR foodborne pathogens highlights the critical need for effective, natural, and consumer-acceptable antibacterial methods. EOs and bacteriophages, each with unique modes of action, have shown great promise as biocontrol agents. When employed in conjunction, they provide a potentially synergistic approach that takes advantage of EOs’ broad-spectrum action and membrane-disruptive properties, as well as bacteriophages’ selectivity and self-replication. This review focuses on new evidence supporting the efficacy of such combinations, such as improved bacterial elimination, reduced resistance development, and applicability in a variety of food matrices. However, the obtained results are still highly dependent on environmental variables, formulation procedures, and microbial traits. Importantly, the interaction between EOs and phages is not always synergistic, and must be carefully controlled to avoid neutralization or antagonistic effects. Future studies should prioritize standardized evaluation protocols, validation in complex food models, development of advanced delivery systems (e.g., encapsulation), and omics-based approaches to better understand molecular interactions. EO–phage combinations are particularly well suited to food systems, due to their complementary strengths. While EOs can impair bacterial defenses even within complex food matrices, bacteriophages offer strain-specific action without affecting beneficial microbiota. This synergy enables effective pathogen control at lower EO concentrations, minimizing sensory impacts on the food product. The natural origin and safety profile of EOs and bacteriophages further support consumer acceptance, especially within clean-label and minimally processed food trends. Furthermore, addressing regulatory frameworks and assessing consumer acceptance will be essential for translating these findings into practical, industry-scale applications. If these challenges are successfully addressed, the combination of EOs and bacteriophages could represent a powerful, clean-label solution for next-generation sustainable food preservation.

## Figures and Tables

**Table 2:** Summary of studies evaluating the combined antimicrobial effects of essential oils and bacteriophages against foodborne pathogens.

Study (Author, Year)	Target Pathogen	Essential Oil	Phage Type	Food Matrix	Method	Main Outcome
Ghosh, 2015 [91]	*S. aureus* (incl. MRSA)	Lemongrass, cinnamon, melissa, tea tree	Phage K	Raw chicken meat	In vitro + inoculated food	No synergy at 6–13 °C; higher phage effect at 25 °C
Ghosh et al., 2016 [92]	*S. aureus* (incl. MRSA)	EO compounds (alpha-pinene, 3-carene)	Phage K	In vitro	Disk diffusion	Additive/synergistic inhibition at high EO conc. (3.28%)
Abdallah et al., 2021 [51]	*S. aureus* (MDR)	Thyme oil (0.5–1%)	vB_SauM_CP9 (*Myoviridae*)	Chicken fillets	Surface application	Synergistic reduction (87.2%) after 120 min
Kim et al., 2024 [49]	*S. typhimurium*	Carvacrol, eugenol, thymol (½ MIC), AITC (allyl isothiocyanate)	Phage endolysin (LysPB32)	Cooked ground beef	In vitro + food trial	>2-log CFU/g reduction in meat; synergistic membrane disruption
Elafify et al., 2025 [93]	*E. coli* ATCC 15597	Cinnamon, thymol (½ MIC)	MS2 + T7 cocktail	In vitro	Spot test, time-kill assay, fitness/mutation assays	Strong synergy; >5-log reduction; reduced resistance

## Data Availability

No new data were created or analyzed in this study. Data sharing is not applicable to this article.

## Abbreviations

The following abbreviations are used in this manuscript:

EOs	Essential oils
AMR	Antimicrobial resistance
MDR	Multidrug-resistant
ROS	Reactive oxygen species
FDA	Food and Drug Administration
GRAS	Generally Recognized as Safe
RTE	Ready-to-eat
EU	European Union
EFSA	European Food Safety Administration
US	United States
CFR	Code of Federal Regulations
GRN	Generally Recognized as Safe
EPA	United States Environmental Protection Agency
FCN	Food Contact Notification
USDA	United States Department of Agriculture
FSIS	Food Safety and Inspection Services
FSANZ	Food Standards Australia New Zealand
BAG	Swiss Federal Office of Public Health (BAG)
CRISPR	Clustered Regularly Interspaced Short Palindromic Repeats
FIC	Fractional Inhibitory Concentration
